# Supplementary material for: A Physiological Approach to Explore How Thioredoxin–Glutathione Reductase (TGR) and Peroxiredoxin (Prx) Eliminate H2O2 in Cysticerci of Taenia
Source: Antioxidants (Basel). 2024 Apr 10;13(4):444. doi: 10.3390/antiox13040444 (PMC11047392; doi:10.3390/antiox13040444)
Supplement: Supplementary file 1 [file antioxidants-13-00444-s001.zip › antioxidants-2879030-supplementary.pdf]

Article

A physiological approach to explore how thioredoxin-glutathione reductase (TGR) and peroxiredoxin (Prx) eliminate H_2O_2 in cysticerci of *Taenia*

Alberto Guevara-Flores[1], Gabriela Nava-Balderas*[2], José de Jesús Martínez-González[1], César Vásquez-Lima[1], Juan Luis Rendón[1] and Irene Patricia del Arenal Mena*[1]

[1]Departamento de Bioquímica, Facultad de Medicina, Universidad Nacional Autónoma de México (UNAM). Apartado Postal 70-159, 04510 Mexico City, Mexico
[2]Departamento de Microbiología y Parasitología, Facultad de Medicina, Universidad Nacional Autónoma de México (UNAM). Apartado Postal 70-159, 04510 Mexico City, Mexico
* Correspondence: gabrielanavab@comunidad.unam.mx (G.N-B) darenal@bq.unam.mx (I.P.d.A).

Supplementary Materials:

A)

Localization	Likelihood
Plastid	0.5505
Mitochondria	**0.4387**
Nucleus	0.0061
Extracelullar	0.0039
Cytoplasm	0.0003
Endoplasmic reticulum	0.0002
Lisosome/vacuole	0.0001
Golgi apparatus	0.0001
Cell membrane	0.0001
Peroxisome	0.0000

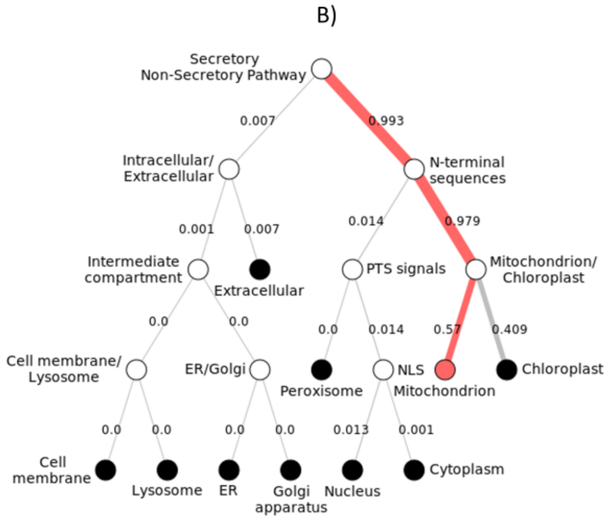

Figure S1. Prediction of *TsPrx3* subcellular localization. Peptide (–MQRLMPHLRPKLFASL-SASSHIAPTFQSR-) of *Ts*Prx3 was analyzed using DeepLoc-1.0. (A) Table of predicted subcellular localization. (B) Hierarchical tree.

Table S1. Kinetic constants for *Ts*TGR and *Tc*TGR toward recombinant Trx from *T. solium* and Trx from *T. crassiceps*.

Enzyme	Oxidizing substrate	K_m	k_{cat}	k_{cat}/K_m	Reference
		**(M)**	**(s^{-1})**	**(M^{-1} s^{-1})**	
*Tc*TGR	*Ts*Trx	41.5 ± 5.3 x 10^{-6}	53.3 ± 4.0	1.2 x 10^{6}	This work*
*Ts*TGR	*Ts*Trx	27.4 ± 3.5 x 10^{-6}	20.0 ± 1.2	0.7 x 10^{6}	[38]
*Tc*TGR	*Tc*Trx	3.1 ± 1.0 x 10^{-6}	10	3.2 x 10^{6}	[43]

*Data obtained using 100 μM NADPH (reducing substrate), 56 nM *Tc*TGR, 50 μM insulin, and increasing concentrations of Trx from *T. solium* at 25°C and pH 7.8. Data are the means of three independent measurements obtained from the Michaelis-Menten equation.

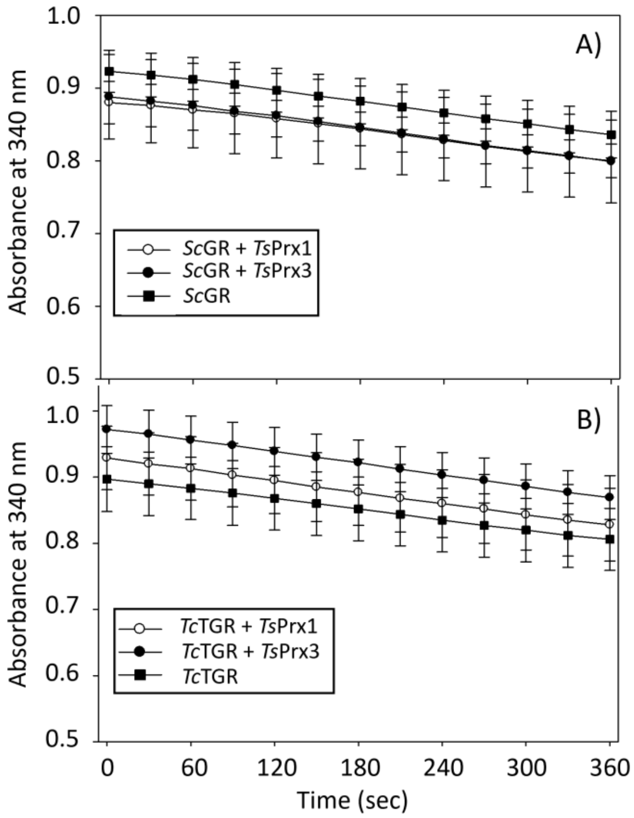

Figure S2. Activity of *Ts*Prx1 and *Ts*Prx3 was determined with the glutathione system. Assays containing 1 mM GSH, 150 μM NADPH, 1.25 μM *Ts*Prx1 (circles) or *Ts*Prx3 (filled circles) were incubated with: (A) 8 nM *Sc*GR (squares) or (B) 11.2 nM *Tc*TGR (squares) in 100 mM Tris/HCl (pH 7.8) 1 mM EDTA for 5 min to allow the reaction to stabilize. H_2O_2 (50 μM) was added to initiate the reaction that was monitored for 6 min at 25°C.

Table S2. Kinetic constants for recombinant *Ts*Prx1 and *Ts*Prx3 toward hydroperoxides in the presence of *Ts*Trx.

		**Oxidizing substrates**					
**Enzyme**	**Reducing Substrate**	**Cumene hydroperoxide**			***t*-butylhydroperoxide**		
		K_m (M)	k_{cat} (s^{-1})	k_{cat}/K_m ($M^{-1}\ s^{-1}$)	K_m (M)	k_{cat} (s^{-1})	k_{cat}/K_m ($M^{-1}\ s^{-1}$)
*Ts*Prx1	*Ts*Trx	$5.1 \pm 0.9 \times 10^{-6}$	$21 \pm 2.0 \times 10^{-3}$	0.4×10^{4}	$18.1 \pm 6.8 \times 10^{-6}$	$6.8 \pm 0.8 \times 10^{-3}$	0.3×10^{3}
*Ts*Prx3	*Ts*Trx	$6.3 \pm 2.6 \times 10^{-6}$	$24 \pm 5.0 \times 10^{-3}$	0.3×10^{4}	$8.4 \pm 2.7 \times 10^{-6}$	$14.0 \pm 1.0 \times 10^{-3}$	1.6×10^{3}

Measurements obtained as described under Materials and Methods at 25°C and pH 7.8. Data are from three independent measurements.

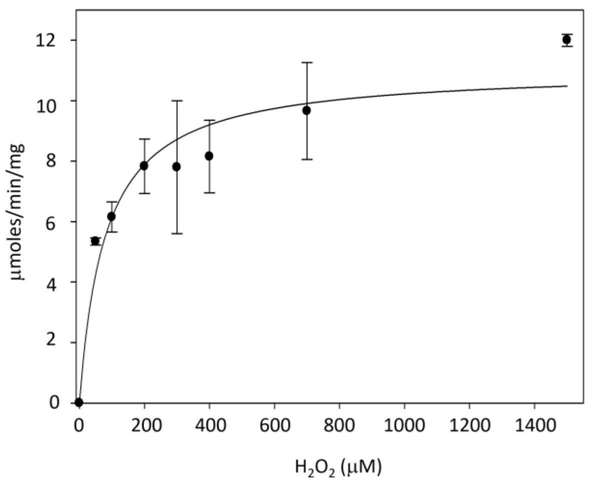

Figure S3. Thioredoxin peroxidase activity of *Tc*TGR. H_2O_2 reduction was measured in the absence of *Ts*Prx1. Measurements obtained as described under Materials and Methods. Data are the means of three independent measurements.

References

38. Nava, G.; Maldonado, G.; Plancarte, A. Cloning, expression, purification, and kinetic characterization of mitochondrial thioredoxin (TsTrx2), cytosolic thioredoxin (TsTrx1), and glutaredoxin (TsGrx1) from *Taenia solium*. *Parasitol Res*. **2019**, *118*, 1785-1797. https://doi.org/10.1007/s00436-019-06336-4

43. Martínez-González, J.J.; Guevara-Flores, A.; Rendón, J.L.; Sosa-Peinado, A.; Del Arenal Mena, I.P. Purification and characterization of *Taenia crassiceps* cysticerci thioredoxin: insight into thioredoxin-glutathione-reductase (TGR) substrate recognition. *Parasitol Int*. **2015**, *64*,194-201. https://doi.org/10.1016/j.parint.2014.12.004
